# War and Peace

**Published:** 1905-10

**Authors:** 


A Monthly Review of Medicine and Surgery.

*EDITOR:*

WILLIAM WARREN POTTER, M. D.

All communications, whether of a literary or business nature, books for review and exchanges, should be addressed to the editor 284 FRANKLIN ST., BUFFALO, N. Y.

VOL. LXI. OCTOBER, 1905. NO. 3

### War and Peace.

WHEN Vice-Admiral Togo, commanding the forces of the Imperial Japanese Navy, on February 8, 1904, opened his guns upon the land and water defences of Port Arthur, the detonations reverberated around the world. This audacity astonished the nations of the earth. Not that rumors and threatenings of war had not filled the atmosphere, but it was hoped, not to say believed, that some compromise would be made and war averted. But the arrogance of Russia, which had dominated the East and defied the powers of continental Europe so long, would not yield to the equities presented by Japan,—and the war came!

The causes of the stupendous contest between the Goliath of the North and the David of the island empire may be briefly stated as follows:

> First—Russia's policy of playing fast and loose with her promises as to the evacuation of Manchuria, whereby China's sovereignty over that land was practically annulled, and the equal commercial rights therein of the rest of the world seriously threatened. Second—Russia's refusal to recognise Japan's paramount interests in Corea, and her own "diplomatic" moves in that peninsula looking toward treaty rights under which she might gain control of the port of Fusan; the more northern harbors of Vladivostok and Port Arthur having proved less serviceable than had been anticipated. Third—Japan's seven-year-old grudge against Russia for ousting her from Port Arthur at the close of her contest with China, and also Russia's fatal ignorance of Japan's preparedness as well as her over-confidence in her own strength.

Chronologically speaking, the principal events after Togo's introductory salutes on February 8 and 9, during which Russia lost two vessels sunk and seven disabled, and the Japanese, with the loss of but two torpedo boats, took command to the Far Eastern Waters, progressed in the following order: May 1, the Yalu river; May 26, Nan-Shan hill and Kin-Chow; June 14-15, Wa-fang-Kao; August 10, attempt of the Russian fleet to escape from the clutches of Togo at Port Arthur, during which the Japanese scattered and disabled the Russian vessels so they ceased, from that time, to be a factor of moment; August 26 to September 4, Liao-Yang; October 11 to 21, Sha river; November 30, 203-Meter hill, the bloodiest of the siege encounters before Port Arthur and which led to its surrender January 2, 1905. The Nun river or Sandepas battle, January 25 to 29, was a preliminary struggle, leading up to the greatest of land battles—Mukden, February 24 to March 12; finally, the crowning glory of the Japanese armies occurred in the battle of the Sea of Japan May 27-28, when Admiral Togo annihilated the combined fleets under Rojestvensky and dismissed Russia from the waters of the Far East.

Togo's guns had hardly more than cooled off when talk of peace began to attract attention in the newspapers and in diplomatic and other government circles all over the civilised world, for it was apparent that further contest on the part of Russia was useless. Beaten everywhere on land and sea without a single engagement to her credit, further resistance seemed hopeless.

President Roosevelt seized a favorable opportunity early in June to address identical notes to the Czar and the Mikado urging them to appoint plenipotentiaries to negotiate terms of peace. After considerable discussion the President's proposition was agreed to and Baron Komura and Minister Takahira were designated by the Emperor of Japan, and Baron Rosen and Mr. Sergius Witte by the Emperor of Russia to meet in the United States. Portsmouth instead of Washington, was designated as the place for the conference, the summer heat of the Capital being too extreme.

The plenipotentiaries met August 5 on board the government yacht "Mayflower," and were introduced to each other by the President, with whom they had an informal luncheon. They then proceeded to Portsmouth where the first conference was held on Wednesday, August 9, 1905. After four weeks of strenuous labor on the part of the plenipotentiaries and their respective suites, a treaty of peace finally was signed September 5, 1905, which will be known in history as the "Treaty of Portsmouth." There were but two items in the Japanese bill of particulars that met with serious opposition from the Russians:—those were the reclamation of the Saghalien Island, and reimbursement of war expenditures.

Baron Komura finally withdrew the indemnity item, whereupon M. Witte, whose shibboleth had been "not a kopek", conceded to Japan the southern half of Saghalien, and the war was ended!

The Japanese nation is to be congratulated in pursuing to a complete finish, with victory everywhere on land and sea, a war with a power never before vanquished, and one that had become so dogmatic, imperious, and offensive in its boastful swagger, that the people of the earth had come to believe it invincible. However, the world also is to be congratulated that peace has come through the valor of this comparatively small nation, in a war waged for a principle and in which every point sought has been attained.

